# Researching Parental Socialization Styles across Three Cultural Contexts: Scale ESPA29 Bi-Dimensional Validity in Spain, Portugal, and Brazil

**DOI:** 10.3390/ijerph16020197

**Published:** 2019-01-11

**Authors:** Isabel Martínez, Fernando Garcia, María C. Fuentes, Feliciano Veiga, Oscar F. Garcia, Yara Rodrigues, Edie Cruise, Emilia Serra

**Affiliations:** 1 Department of Psychology, University of Castilla-La Mancha, Avda de los Alfares 44, 16071 Cuenca, Spain; 2 Department of Methodology of the Behavioral Sciences, University of Valencia, Av. Blasco Ibanez, 21, 46010 Valencia, Spain; fernando.garcia@uv.es (F.G.); m.castillo.fuentes@uv.es (M.C.F.); 3 Instituto de Educação, Universidade de Lisboa, Alameda da Universidade, 1649-013 Lisboa, Portugal; fhveiga@ie.ulisboa.pt (F.V.); yaraiglesia@gmail.com (Y.R.); 4 Department of Developmental and Educational Psychology, University of Valencia, Av. Blasco Ibanez, 21, 46010 Valencia, Spain; oscar.f.garcia@uv.es (O.F.G.); emilia.serra@uv.es (E.S.); 5 Department of Business Administration, University of Trier, Universitätsring 15, D-54296 Trier, Germany; edie.cruise@gmail.com

**Keywords:** parenting styles, parental warmth and strictness, adolescents, factorial invariance, multi-group analysis

## Abstract

Recent research that relates parenting with adolescent adjustment has shown the importance of considering the cultural context of the relationship. New results are emerging when considering the classical four-typologies model of parental socialization in some European and South-American countries. Among the instruments used in this emergent research is the Parental Socialization Scale ESPA29. This scale is a bi-dimensional parenting instrument that was specifically developed to measure the four parenting typologies, through the dimensions of acceptance/involvement and strictness/imposition. This study examines the good fit of the orthogonal bi-factor model based on the ESPA29 versus one-dimensional and bi-dimensional oblique alternative models, with three adolescent samples from 12 to 17 years old (53.4% girls), from Spain (*N* = 826), Portugal (*N* = 752), and Brazil (*N* = 628). We applied structural equation models (SEMs) to analyze the fit of the models to the data. The results confirm a better fit to the data for the orthogonal bi-factor model versus one-dimensional and bi-dimensional oblique alternative models across country, adolescent sex, and the three age groups. Additionally, the convergent validity of the scale was proved by showing the relation of the two parenting dimensions with self-concept. The results guarantee the adequacy of the ESPA29 to measure parenting styles.

## 1. Introduction

Research on parental socialization has coincided in pointing out two dimensions of parenting behavior. Although the labels utilized to denominate the dimensions have varied since the work of Maccoby and Martin (1983) [1] they have frequently been denominated as demandingness and responsiveness [2]. The demandingness dimension represents to what degree parents supervise and demand maturity of their children, assertively uphold their authority and use control over their children. The responsiveness dimension refers to the extent to which parents demonstrate emotional warmth, such as affection, and acceptance to their children, support them and utilize reasoning in their communication with them [3,4].

Earlier scholars utilized other labels such as control (Watson, 1928) [5] or attachment (Freud, 1933; Rogers, 1960) [6,7] to define the two main parenting dimensions. Symonds (1939) [8] used the terms acceptance/rejection and domination/submission, whereas Baldwin (1955) [9] named them emotional warmth/hostility and indifference/commitment. In the same line, Schaefer (1959) [10] named the two dimensions love/hostility and autonomy/control, while Sears, MacCoby, and Levin (1957) [11] used the labels of warmth and permissiveness/inflexibility, and Becker (1964) [3] talked about warmth/hostility and restriction/permissiveness. Baumrind [12,13,14,15] also confirmed two underlying dimensions in parent–child relationships named acceptance and paternal control. Later, in the work carried out by Steinberg and colleagues (1994) [16], two dimensions with similar connotations were identified: Acceptance/involvement and strictness/supervision [17,18]. Acceptance/involvement and strictness/imposition (ESPA29, Musitu & García, 2001) [19], have also been utilized in different recent works [20,21,22]. To sum up, these two central parenting dimensions represent two different and theoretically unrelated parental behavior patterns [23] that when considered together lead to the four parental socialization styles: Authoritative—high use of demandingness parenting behaviors and high use responsiveness behaviors; neglectful—low use of both dimensions; indulgent—low use of demandingness and high use of responsiveness; and authoritarian style—high use of both dimensions [1,18,24]. Responsiveness has often been measured through parental warmth and acceptance, while demandingness has been operationalized as parental firmness [2].

The Parental Socialization Scale ESPA29 [19] is a bi-dimensional parenting instrument that was created with the precise purpose of measuring the aforementioned parenting typologies. The four parenting typologies are measured through the dimensions of acceptance/involvement and strictness/imposition, which are considered independent. The questionnaire specifically considers the distinction between socialization practices and styles [23,25,26] using a contextual [23] and situational [26] approach. The ESPA29 analyzes behaviors showed by parents in specific situations that delineate day-to-day life within a family in Western culture. The instrument inquires about parental behavior within said situations through questions posed to the adolescent. The scale measures the use that mothers and fathers make of seven different practices of socialization: Warmth, indifference, reasoning, detachment, verbal scolding, physical punishment, and revoking privileges. The acceptance/involvement dimension consists of the practices of warmth and reasoning that compose the positive pole of the dimension, whereas indifference and detachment practices form the negative pole. The strictness/imposition dimension is formed with the verbal scolding, physical punishment, and revoking privileges practices. The practices that make up the two dimensions do not relate to each other; the strictness/imposition practices are impositive practices that are independent of the degree of acceptance/involvement. In this way, the possibility of a parent using an acceptance/involvement practice, such as reasoning, following the use of a strictness/imposition practice, such as scolding or revoking priveleges, is accounted for, as well as the possibility of a parent choosing to use only one of these practices of the two dimensions. The four parenting styles—authoritative, authoritarian, indulgent, and neglectful—are formed through the scores obtained on parental behavior comprising the acceptance/involvement and strictness/imposition dimensions.

The ESPA29 has been utilized to relate parenting with a wide variety of variables that capture adolescent adjustment using the four parenting styles [1,27,28,29] or the two main dimensions of parenting [30,31,32]. Among the adolescent adjustment criteria utilized are self-esteem [4], personal values [33], academic engagement [34], bullying and cyberbullying involvement [35], substance use [36], and antisocial behavior [37,38]. The instrument has been used mainly in Spain [31,33,37,39] but also in other countries like Portugal [40], Brazil [4,41], the United States [30], Italy [42], and Peru [43]. The ESPA29 scale is among the instruments used in emergent research that question authoritative parenting as the optimal style of socialization in any culture. Studies recently carried out in Europe and Latin America, namely in Spain [44], Italy [45], UK, Sweden, Slovenia, Czech Republic [46], Norway [22], Germany [47], Portugal [40], Turkey [48], Brazil [49], and Mexico [50], coincide in finding that, in those cultural contexts, the indulgent parenting style relates to equal or even higher adolescent adjustment than authoritative parenting.

The theoretical factor structure of the ESPA29 has been confirmed by the Exploratory Factor Analyses (EFAs) in Spain, where the scale was originally developed [19]. Subsequently, the factor structure has been confirmed in other languages and countries, including the Basque Country [51], Italy [42], and Brazil [41,52], using EFA and Procrustes Rotations [53]. The concurrent validity of the ESPA29 has also been successfully tested with two different samples from Spain [27,54]. Finally, Confirmatory Factor Analysis (CFA) was applied in the validation of the ESPA29 in a sample from the United States [30], although CFA has not yet been applied in Spain or any other country where the instrument has been used. Furthermore, the better fit of the two dimensions of the scale in an orthogonal model in comparison to oblique or one-dimensional models has not been confirmed.

The present study has two objectives. The first is to analyze the orthogonal bi-factor model based on ESPA29 as compared to one-dimensional and bi-dimensional oblique alternative models with three adolescent samples, one Spanish, one Portuguese, and another Brazilian. The second objective is to examine the invariance of the orthogonal bi-factor model based on the ESPA29 with the three samples of Spanish, Portuguese, and Brazilian adolescents. It is hypothesized that: (1) The bi-factor orthogonal model will provide a better fit to the data than the two alternative models; and (2) the adjustment of Spanish, Portuguese, and Brazilian samples will be invariant with respect to country, sex, and age.

Additionally, to test the convergent validity of the scale, the two dimensions—acceptance/involvement and strictness/imposition—will be related to adolescent self-esteem, a classic criteria variable in parental socialization studies [33,55,56]. According to previous research [31,57], it is hypothesized that the practices of acceptance/involvement will relate positively to self-esteem, whereas the strictness/imposition dimension will relate negatively.

## 2. Materials and Methods

### 2.1. Participants

The sample was composed of 2,207 adolescents (53.4% being women, 37.4% Spanish, 34.1% Portuguese, and 28.5% Brazilian) covering the adolescent age range of 12 to 17 years old (*M* = 14.12, *SD* = 1.67) (see Table 1).

### 2.2. Procedure

Our sample was drawn from students attending educational centers from urban areas with a population of over one million in the three cities where the study was carried out, situated on the East Coast of Spain, the Middle West Coast of Portugal, and in the Southeast of Brazil. The data were collected from 16 secondary schools (5 Spanish, 5 Portuguese, and 6 Brazilian) chosen at random utilizing the simple random sampling method from a comprehensive list of those cities’ schools.

We obtained approval to conduct this research through the Valencian Research Ethics Committee of the Program for the Promotion of Scientific Research, Technological Development and Innovation in Spain. After that, it was necessary for each of the Research and Evaluation Boards in the cities where the study was carried out to approve this research. After having obtained their approval, we were then allowed to conduct the study in the individual secondary schools by the head or principal of each educational center. The next step of approval was then granted by each teacher or instructor for our questionnaires to be completed during their class time. Our team informed each student and their parents or legal guardians of the nature of our study through a letter, which was then signed by both a parent/guardian and the student, ensuring we were granted permission from a parent/guardian, as well as assent from the student agreeing to partake in the research voluntarily. The anonymous questionnaires were only administered to those students who agreed to complete it and had parental/guardian permission to do so. We examined the questionnaires for aberrant response patterns, such as reporting implausible inconsistencies between negatively and positively worded responses or “maximum-scale” behavior [44,57,58,59]. About 4% (*n* = 83) of the cases contained such inconsistencies and were therefore eliminated from the sample.

### 2.3. Instruments

#### 2.3.1. Parental Socialization

The Parental Socialization Scale ESPA29 [19] is a self-report instrument, designed to examine parenting styles via children’s and adolescents’ responses, aged 10 to 18 years. This instrument measures distinct parenting practices in the context of day-to-day family life. These specific parenting practices are measured as responses to 29 situational contexts which are common occurrences between adolescents and their parents. Within the 29 situations, there are 13 which give the context of obedience in which the family norm is followed (e.g., “If I bring home my report card with good grades”) and 16 which portray a context of disobedience in which the family norm is contravened (e.g., “If they find out that I have lied”). The parenting practices of warmth (“He/she shows affection”) and indifference (“He/she seems indifferent) are measured in response to the 13 contexts of obedience while the parenting practices of reasoning (“He/she talks to me”), detachment (It’s the same to him/her”), verbal scolding (“He/she scolds me”), physical punishment (“He/she hits me”), and revoking privileges (“He/she takes something away from me”) are measured in response to the 16 contexts of disobedience. The adolescent respondent uses a 4-point scale to indicate the frequency in which their mother and father make use of the seven specified parenting practices, with 1 meaning “never”, 2 “sometimes”, 3 “most times”, and 4 “always”.

To calculate the score of the acceptance/involvement dimension, the scores of the detachment and indifference subscales are first inverted given their negative relation to the dimension. Then, the scores of warmth, reasoning, indifference, and detachment subscales can be averaged to produce the aggregate score for the dimension. Similarly, the strictness/imposition dimension score is also comprised of an average of the revoking privileges, verbal scolding, and physical punishment subscales. No inversion is necessary in this case as all three subscales relate positively to the dimension. The aggregate dimension scores for each sample across country, sex, and age group can be found in Table 1.

The instrument needed to be translated from Spanish into Portuguese in order to carry out this study. We first obtained permission from the scale’s authors to do so and then selected three bilingual (Spanish- and Portuguese-speaking) colleagues to perform the Spanish to Portuguese translation. The bilingual team verified equivalence in grammar, clarity, and content item by item. Once that was completed, a back-translation was performed by an additional bilingual researcher independent from the present study. Finally, the scale’s authors reviewed the back-translated Portuguese to Spanish version for final verification and approval [41,60].

#### 2.3.2. Multidimensional Self-Concept

The AF5 [61,62] measures self-concept through five dimensions: Academic (e.g., “I work very hard in class”), social (e.g., “I make friends easily”), emotional (e.g., reversed item, “It is difficult for me to talk to strangers”), family (e.g., “I am happy at home”), and physical (e.g., “I take good care of my physical health”). There is a total of 30 items that comprise the scale divided into six per dimension. The participant rates the items, which are statements, according to his/her level of agreement or disagreement using a 99-point scale (portrayed by a thermometer), which ranges from 1, representing complete disagreement, to 99, representing complete agreement.

The factor structure of the AF5 was confirmed with exploratory and confirmatory analyses [57,58,59,60,61,62,63,64,65] and no method effect appears to be associated with negatively-worded items [58,59]. The instrument was originally developed and validated in Spain [61] and has also been validated in English [60], Basque [64], and Catalan languages [65]. Numerous studies have utilized the AF5 to relate self-esteem to other variables (e.g., gender stereotypes, body image, and sport practice [66], physical activity [67], motivational climate [68], food neophobia [57], substance use [69,70,71], participation in school violence [37], and subjective well-being [72]) with consistent results. Lastly, higher adolescent self-esteem has been found to be related to the ESPA29 dimension of parental acceptance/involvement, whereas lower adolescent self-esteem has been related to the strictness/imposition dimension in different studies [30,31,57].

### 2.4. Data Analysis

We began by examining how well the theoretical orthogonal two-factor model of socialization fit the data against two alternative models. We first tested a one-factor model, which conceives parenting as a one-dimensional construct (e.g., one-dimensional parental acceptance-rejection socialization theory [73]). Next, we tested the oblique (correlated) two-factor model, whereby parenting is as a bi-dimensional construct in which parental acceptance/involvement and parental strictness/imposition are correlated [25,46,74]. Third, we tested the theoretical orthogonal two-dimensional model. Under this model, parenting is conceived as a bi-dimensional construct where the underpinning parenting dimensions are unrelated or orthogonal. In this model, we free the covariate between the two factors of the bi-factor model. This theoretical orthogonal bi-factor model is the same model as the previous oblique one but with the two dimensions non-correlated [23,26,27,30]. We freed error covariances for the strongly correlated pairs of parenting practices whose content was more alike [30,57,75,76].

In order to analyze the fit of the models to the data, we calculated structural equation models (SEMs) using EQS 6.1 (Multivariate Software, Encino, CA, USA) [9]. We employed the maximum likelihood robust estimation method due to the deviation of the multinormal data (all Mardia’s normalized coefficient >25, *p* < 0.01). In order to control non-normality, the scale of parenting practices was transformed into quartiles [59,77], the correlation matrices used were polychoric, and the models were tested with the Satorra-Bentler chi-squared statistic [78] and associated robust confirmatory fit index provided by EQS 6.1 [9]. The criteria used are in line with those proposed by Hu and Bentler [79] and are the usual criteria utilized in this type of analysis [30,57].

The CFA technique allows for the adjustment of the model to the data to be evaluated through the chi-squared value obtained. However, the chi-squared test has shown serious problems of sensitivity to sample size [21,80,81]. Methodological studies provide other fit indexes which have the advantage of a pre-established cut-off criteria [30,60,63,81]. We applied the following indexes: Root mean squared error of approximation (RMSEA), where values lower than 0.08 are considered acceptable; normed fit index, incremental fit index, and comparative fit index, NFI, IFI, and CFI, whose value must exceed 0.90; and the information criterion of Akaike, AIC (Akaike information criterion), where the lowest value indicates the highest parsimony [82]. RMSEA too often falsely indicates a poorly fitting model for small *df* models [83], i.e., one-dimensional and two-dimensional parenting practices models.

To test the second hypothesis—the invariance of the country, sex, and age sample—we evaluated four nested models that progressively increased the number of restrictions by constraining free parameters. After establishing what the model baseline was, we conducted the following sequence of increasingly more restrictive tests of invariance across the three samples: Model A, unconstrained, without any restrictions across any parameters for the thee samples examined; Model B, we fixed factor pattern coefficients; Model C, we fixed factor variances and covariances; and Model D, finally, we established the equality of the error variances. At each step, when the parameters of the previous model are restricted, the degrees of freedom of the new model increase and chi-square also tends to increase. When Δχ2 value is statistically significant, the null hypothesis that the models are equivalent to, it rejects. Cheung and Rensvold (2002) [81] provided a solution to the oversensitivity problem of Δχ2 to sample size by examining the invariance of nested models via the ΔCFI. After analyzing 20 different adjustment indexes, these authors (2002, p. 251) [81] concluded that an absolute ΔCFI value higher than 0.01 (i.e., |ΔCFI| > 0.01) signifies a meaningful fall in fit.

## 3. Results

### 3.1. Fitting of Model to Data from the One-Dimensional to Two-Dimensional Orthogonal Model

First, we constrained the data to test their adjustment with the one-dimensional model (Table 2). The statistics produced from that calculation did not reach cut-off values, resulting in a poor fit of the model to the data (father, RMSEA = 0.20, CFI = 0.80, IFI = 0.80, NFI = 0.80, AIC = 710; mother, RMSEA = 0.18, CFI = 0.82, IFI = 0.82, NFI = 0.81, AIC = 566). Second, we constrained the data to test their adjustment with the two-dimensional oblique model, which resulted in a significantly improved fit against the previous model (father, RMSEA = 0.10, CFI = 0.96, IFI = 0.96, NFI = 0.96, AIC = 144; mother, RMSEA = 0.11, CFI = 0.96, IFI = 0.96, NFI = 0.96, AIC = 163). Finally, we constrained the data to test their adjustment with the theoretical orthogonal model, which did not yield a fall in fit compared to the oblique model (father, RMSEA = 0.10, CFI = 0.95, IFI = 0.95, NFI = 0.95, AIC = 160; mother, RMSEA = 0.08, CFI = 0.97, IFI = 0.96, NFI = 0.96, AIC = 100), although the orthogonality restriction has been included by fixing the covariation between the two factors to 0 (i.e., Acceptance/involvement and strictness/imposition).

### 3.2. Multi-Group Confirmatory Factor Analyses of Invariance

Multi-group confirmatory factor analyses of invariance across country, age, and sex groups are reported in Table 3. The unconstrained parsimoniously orthogonal model indicated a good fit, suggesting a common factor structure across country, sex, and age groups. Constraining the measurement weights, structural variances, and covariances, and measurement residuals yielded non-significant changes in fit across country, sex, and age groups, |ΔCFI| <0.01.

Table 4 gives an overview of the factor loadings estimated in the most constrained model. Invariance testing across language, sex, and adolescent age indicated analogous functioning of the orthogonal bi-factor model in all of the samples examined.

Additionally, we calculated the two parenting dimensions, acceptance/involvement and strictness/imposition, with raw data. Father parenting practices were modestly correlated, *r* = 0.16, *R*^2^ = 0.02 (2%), *p* < 0.01. Neither the 95% CI (0.12, 0.20) nor the 95% CI proportion of variance (0.01, 0.04) included zero. In the same line, mother parenting dimensions were also modestly correlated, *r* = 0.09, *R*^2^ = 0.01 (1%), *p* < 0.01. Although the 95% CI (0.09, 0.05) did not included zero, the 95% CI proportion of variance (0.00, 0.02) did include zero.

### 3.3. Reliability

Father alpha reliability coefficients for the total scale were 0.93, in the Spanish sample, 0.92, in the Portuguese, 0.93, in the Brazilian, 0.93, in women, 0.93, in men, 0.93, in the 12–13-year-old age group, 0.93, in the 14–15-year-old age group, 0.92, and in the 16–17-year-old age group, 0.92. Mother alpha reliability coefficients for the total scale were 0.93, in the Spanish sample, 0.93, in the Portuguese, 0.93, in the Brazilian, 0.91, in women, 0.93, in men, 0.93, in the 12–13-year-old age group, 0.93, in the 14–15-year-old age group, 0.92, and in the 16–17-year-old age group, 0.92 (see Table 1).

### 3.4. Relation with Self-Concept Dimensions

Regarding the relation between the ESPA29 acceptance/involvement dimension and self-concept, the Pearson correlation revealed that father and mother scales were positively associated with academic, social, family, and physical self-concept. With respect to the strictness/imposition dimension, the father scales showed a negative association with academic, social, emotional, and family self-concept, as well as the mother scales with emotional and family self-concept (Table 5).

The size of the correlations between parental socialization dimensions and self-concept is similar to those reported in previous studies that examine the relation between these two variables [19,30,55,56]. It was noted that family self-concept correlation with acceptance/involvement was 0.42 (*r*^2^ = 18%) for the father and 0.41 (*R*^2^ = 17%) for the mother. Additionally, strictness/imposition correlation with family self-concept was −0.33 (*R*^2^ = 11%) for the father and −0.16 (*R*^2^ = 3%) for the mother [19,84]. In addition, it was noted that strictness/imposition correlation with emotional self-concept was −0.18 (*R*^2^ = 3%) for the mother.

## 4. Discussion

The results of this work confirm the orthogonal bi-dimensional structure of the Parental Socialization Scale ESPA29 [19] with three samples of adolescents from Spain, Portugal, and Brazil. Confirmatory Factor Analyses confirm a better fit to the data of the orthogonal bi-factor model as compared to competitive one-dimensional and bi-dimensional oblique alternative models of parenting across country (Spain, Portugal, and Brazil), adolescent sex, and three age groups from 12–17 years old. These results are consistent for both fathers’ and mothers’ scores, supporting the two dimensions of parental conduct proposed in the ESPA29, where the dimension of acceptance/involvement is measured with the warmth and reasoning subscales, which loaded positively onto the dimension, and indifference and detachment subscales, which loaded negatively. Meanwhile, the subscales of physical punishment, verbal scolding, and revoking privileges loaded positively onto the strictness/imposition dimension. Furthermore, combined multi-sample nested factor analysis showed that the ESPA29 orthogonal bi-dimensional model is largely invariant across related samples of country (Spain, Portugal, and Brazil), sex, and adolescent age for both fathers’ and mothers’ scores.

The results of the study underline the importance of considering parental practices of socialization in two independent, non-related dimensions [1,23,26] in oposition to one-dimensional or two dimensional oblique models. One-dimensional models [73] would only include a part of the total variance, without considering all the variation of the parenting socialization construct. Moreover, oblique models, where the two parenting dimensions are related, do not allow for the proper measurement of the four parenting styles, since the dimensions will not equally represent the different parenting styles that are defined. For example, the strictness dimension is shared by authoritative and authoritarian styles and should equally define both styles, however, “monitoring”, which has been widely used to capture strictness [16,18], has received serious critiques for not equally representing the two styles (authoritative and authoritarian [25,74]). Although monitoring was initially conceptualized as a parenting practice involving active parents’ attempts to watch over children as a resource of firm control or strictness [16,18], researchers have complained that most of the adolescent outcomes that parental monitoring predicts are explained by adolescents’ spontaneous disclosure of information to parents (characteristic of authoritative parenting), but not by parents’ attempts to obtain accurate information (characteristic of authoritarian parenting) [25,27,46,74,85,86,87,88]. 

Therefore, the ESPA29 conforms to the theoretical model of parenting repeatedly identified in the literature during the last ten decades [1,5,8,11], which identifies two main parental dimensions [16,18,20,21]. When these two dimensions are considered together, they make up the classical parenting typology, which establishes four family styles of parenting: Authoritative, authoritarian, indulgent, and neglectful. In this way, the quadripartite model contemplates the differentiation between neglectful and indulgent parenting unlike tripartite models, such as Baumrind’s model [12,13,14,15], which ignores variations in warmth among families characterized by low levels of control. In doing so, tripartite models use a single category labeled ‘permissive’ to describe these two parenting groups (Lamborn et al. 1991, p. 1050)”.

Additionally, the convergent validity of the scale in those samples was proved by showing the relation of the two parenting dimensions with self-concept, a classic criteria variable in parenting studies [1,16,18,20,21]. The results show that the acceptance/involvement dimension is positively related with self-esteem for mothers’ and fathers’ scores, whereas the strictness/imposition dimension is negatively-related with adolescents’ self-esteem for mothers’ and fathers’ scores. Our results are like those reported in other studies which examine the parenting and self-esteem relationship [89] in that positive parenting is associated with high self-esteem, whereas negative parenting is associated with low self-esteem [16,18,46]. Futhermore, similar results are reported in other studies using the ESPA29 [27,30,31].

This article is not without limitations. Fathers’ and mothers’ scores were calculated from the adolescents’ responses, though research indicates that adolescent self-reports contribute to our comprehension of the family process in a meaningful way [16], and similar results have been obtained on parenting styles despite different methods of data collection [16,18,28,29]. Second, our results are in the context of three countries (Spain, Portugal, and Brazil), but possible differences must be kept in mind if extrapolating to other countries and cultures. Despite the aforementioned limitations, the present work fully corroborates the bi-dimensional structure of parenting as conceptualized and measured by the ESPA29. 

## 5. Conclusions

The present work reinforces the bi-dimensional structure of parenting. The theoretical structure of the Parental Socialization Scale ESPA29 [19], is confirmed with CFA in three samples from Spain, Portugal, and Brazil. The bi-dimensional orthogonal model results in a better fit as compared to the competitive one-dimensional and bi-dimensional oblique alternative models. The results are consistent across country, adolescent sex, and the three age groups from 12 to 17 years old. Therefore, the results confirm the adequacy of the ESPA29 scale to measure parenting styles.

## Figures and Tables

**Table 1:** Sample distribution of fathers’ and mothers’ parenting practices by country, sex, and age.

Sample	N	Acceptance/Involvement						Strictness/Imposition					
		Min	Max	M	SD	Skew	α1	Min	Max	M	SD	Skew	α1
**Father**													
All	2207	1.00	4.00	3.11	0.53	−0.73	0.96	1.00	3.38	1.72	0.41	0.65	0.93
Spanish	826	1.28	4.00	3.17	0.45	−0.66	0.94	1.00	3.04	1.70	0.37	0.62	0.92
Portuguese	752	1.00	4.00	3.09	0.58	−0.73	0.96	1.00	3.21	1.69	0.43	0.79	0.93
Brazilian	629	1.08	4.00	3.07	0.54	−0.62	0.96	1.00	3.38	1.79	0.41	0.51	0.93
Women	1178	1.00	4.00	3.13	0.55	−0.89	0.96	1.00	3.19	1.68	0.39	0.67	0.93
Men	1029	1.00	4.00	3.09	0.50	−0.50	0.95	1.00	3.38	1.78	0.42	0.63	0.93
12–13	770	1.10	4.00	3.21	0.50	−0.73	0.95	1.00	3.38	1.84	0.43	0.47	0.93
14–15	776	1.00	4.00	3.01	0.54	−0.78	0.96	1.00	3.17	1.71	0.39	0.68	0.92
16–17	661	1.00	3.97	3.02	0.52	−0.68	0.95	1.00	2.88	1.61	0.35	0.29	0.92
**Mother**													
All	2207	1.38	4.00	3.20	0.47	−0.51	0.95	1.00	3.38	1.75	0.40	0.60	0.93
Spanish	826	1.38	4.00	3.18	0.46	−0.53	0.96	1.00	3.06	1.71	0.39	0.62	0.93
Portuguese	752	1.38	4.00	3.24	0.49	−0.61	0.95	1.00	3.38	1.74	0.40	0.77	0.93
Brazilian	629	1.68	4.00	3.18	0.47	−0.37	0.95	1.02	3.15	1.82	0.38	0.39	0.91
Women	1178	1.38	4.00	3.23	0.47	−0.60	0.95	1.00	3.38	1.72	0.39	0.72	0.93
Men	1029	1.38	4.00	3.16	0.47	−0.41	0.94	1.00	3.17	1.80	0.41	0.47	0.93
12–13	770	1.68	4.00	3.30	0.46	−0.47	0.94	1.00	3.38	1.88	0.42	0.47	0.93
14–15	776	1.38	4.00	3.18	0.48	−0.57	0.95	1.00	3.04	1.73	0.39	0.63	0.92
16–17	661	1.38	4.00	3.11	0.47	−0.44	0.94	1.00	3.02	1.64	0.37	0.68	0.92

α, alpha of Cronbach.

**Table 2:** Confirmatory factor analysis of fathers’ and mothers’ parenting practices.

Model	S-Bχ^2^	*df*	CFI	IFI	NFI	AIC	RMSEA [90%CI]
**Father**							
One-dimensional	726.36 **	8	0.803	0.803	0.802	710.36	0.202 [0.189–0.214]
Oblique	157.73 **	7	0.959	0.959	0.957	143.73	0.099 [0.086–0.112]
Orthogonal	176.18 **	8	0.954	0.954	0.952	160.18	0.098 [0.085–0.110]
**Mother**							
One-dimensional	581.83 **	8	0.815	0.816	0.813	565.83	0.180 [0.168–0.193]
Oblique	176.56 **	7	0.957	0.955	0.955	162.56	0.105 [0.092–0.118]
Orthogonal	115.96 **	8	0.965	0.963	0.963	99.96	0.078 [0.066–0.091]

S-Bχ^2^, Satorra–Bentler chi-squared; *df*, degrees of freedom; CFI, comparative fit index; IFI, incremental fit index; NFI, normed fit index; AIC, Akaike information criterion (computed as χ^2^ − 2*df*); RMSEA, root mean squared error of approximation. All indexes are the robust version. In oblique and orthogonal bi-dimensional models, covariation between the residuals errors more correlated were added. ** *p* < 0.01.

**Table 3:** Multi-sample analysis of invariance across country, age, and sex of fathers’ and mothers’ parenting practices.

Model	S-Bχ^2^	*df*	CFI	ΔCFI	IFI	NFI	AIC	RMSEA (90% CI)
**COUNTRY**								
**Father**								
Model A	183.70 **	24	0.953		0.953	0.953	135.70	0.055 (0.048–0.062)
Model B	247.13 **	34	0.945	0.008	0.945	0.937	179.13	0.053 (0.047–0.060)
Model C	274.20 **	32	0.939	0.006	0.939	0.930	21.20	0.053 (0.047–0.059)
Model D	372.89 **	52	0.932	0.007	0.932	0.922	268.89	0.053 (0.048–0.058)
**Mother**								
Model A	146.47 **	24	0.962		0.963	0.956	98.47	0.048 (0.041–0.056)
Model B	163.42 **	34	0.960	0.002	0.960	0.950	95.42	0.042 (0.035–0.048)
Model C	185.17 **	38	0.954	0.006	0.955	0.944	109.17	0.042 (0.036–0.048)
Model D	245.66 **	52	0.951	0.003	0.951	0.939	141.66	0.041 (0.036–0.046)
**SEX**								
**Father**								
Model A	181.80 **	16	0.955		0.955	0.951	149.80	0.069 (0.060–0.078)
Model B	191.84 **	21	0.953	0.002	0.954	0.948	149.84	0.061 (0.053–0.069)
Model C	204.37 **	23	0.951	0.002	0.945	0.945	158.37	0.060 (0.052–0.067)
Model D	239.05 **	30	0.951	0.000	0.951	0.945	1790.05	0.056 (0.050–0.063)
**Mother**								
Model A	127.53 **	16	0.964		0.965	0.960	95.53	0.056 (0.047–0.065)
Model B	137.85 **	19	0.963	0.001	0.963	0.957	99.85	0.050 (0.042–0.058)
Model C	144.22 **	21	0.961	0.002	0.961	0.954	102.22	0.049 (0.041–0.057)
Model D	168.96 **	30	0.962	−0.001	0.962	0.954	108.96	0.046 (0.039–0.053)
**AGE**								
**Father**								
Model A	193.71 **	24	0.954		0.954	0.948	145.71	0.057 (0.049–0.064)
Model B	218.83 **	34	0.950	0.004	0.950	0.941	15.83	0.050 (0.043–0.056)
Model C	234.24 **	38	0.946	0.004	0.947	0.937	158.24	0.048 (0.042–0.054)
Model D	279.11 **	52	0.949	−0.003	0.949	0.939	175.11	0.045 (0.039–0.050)
**Mother**								
Model A	16.70 **	24	0.957		0.958	0.951	112.70	0.051 (0.043–0.058)
Model B	186.32 **	34	0.952	0.005	0.953	0.943	118.32	0.045 (0.039–0.051)
Model C	199.45 **	38	0.949	0.003	0.950	0.939	123.45	0.044 (0.038–0.050)
Model D	249.84 **	52	0.949	0.000	0.950	0.937	145.84	0.042 (0.036–0.047)

S-Bχ^2^, Satorra–Bentler chi-squared; *df*, degrees of freedom; CFI, comparative fit index; IFI, incremental fit index; NFI, normed fit index; AIC, Akaike information criterion (computed as χ^2^ − 2*df*); RMSEA, root mean squared error of approximation. All indexes are the robust version. ** *p* < 0.01. Model A, unconstrained baseline model; model B, measurement weights; model C, structural variances and covariances; and model D, measurement residuals.

**Table 4:** Confirmatory factor analysis (CFA) standardized factor loadings of fathers’ and mothers’ parenting practices of the most constrained model.

Parental Practice	Father			Mother		
	Sex	Country	Age	Sex	Country	Age
**Acceptance/involvement**						
Warmth	0.46 **	0.50 **	0.45 **	0.52 **	0.51 **	0.52 **
Indifference	−0.70 ^a^	−0.71 ^a^	−0.70 ^a^	−0.68 ^a^	−0.69 ^a^	−0.68 ^a^
Detachment	−0.53 **	−0.52 **	−0.53 **	−0.51 **	−0.52 **	−0.51 **
Reasoning	0.81 **	0.80 **	0.81 **	0.74 **	0.74 **	0.74 **
**Strictness/imposition**						
Verbal scolding	0.56 ^a^	0.56 ^a^	0.56 ^a^	0.58 ^a^	0.58 ^a^	0.56 ^a^
Physical punishment	0.49 **	0.46 **	0.49 **	0.47 **	0.48 **	0.53 **
Revoking privileges	0.84 **	0.84 **	0.85 **	0.76 **	0.76 **	0.79 **

^a^ Fixed to 1 during estimation. ** *p* < 0.01.

**Table 5:** Correlations and *R*^2^ between two main parental socialization dimensions with five self-concept dimensions.

Self-concept Dimensions	Acceptance/Involvement		Strictness/Imposition	
	*r* (95% CI)	*R*^2^ (95% CI)	*r* (95% CI)	*R*^2^ (95% CI)
**Father**				
Academic	0.234 (0.194, 0.273)	0.05 (0.04, 0.07) *	−0.143 (−0.184, −0.102)	0.02 (0.03, 0.01) *
Social	0.168 (0.127, 0.208)	0.03 (0.02, 0.04) *	−0.128 (−0.169, −0.087)	0.02 (0.03, 0.01) *
Emotional	−0.011 (−0.053, 0.031)	0.00 (0.00, 0.00) ^+^	−0.034 (−0.076, 0.008)	0.00 (0.01, 0.00) ^+^
Family	0.421 (0.386, 0.455)	0.18 (0.15, 0.21) **	−0.325 (−0.362, −0.287)	0.11 (0.13, 0.08) **
Physical	0.133 (0.092, 0.174)	0.02 (0.01, 0.03) *	−0.092 (−0.133, −0.050)	0.01 (0.02, 0.00) ^+^
**Mother**				
Academic	0.245 (0.205, 0.284)	0.06 (0.04, 0.08) *	0.018 (−0.024, 0.060)	0.00 (0.00, 0.00) ^+^
Social	0.191 (0.150, 0.231)	0.04 (0.02, 0.05) *	0.011 (−0.031, 0.053)	0.00 (0.00, 0.00) ^+^
Emotional	−0.030 (−0.072, 0.012)	0.00 (0.01, 0.00) ^+^	−0.178 (−0.218, −0.137)	0.03 (0.05, 0.02) *
Family	0.409 (0.374, 0.443)	0.17 (0.14, 0.20) **	−0.160 (−0.200, −0.119)	0.03 (0.04, 0.01) *
Physical	0.135 (0.094, 0.176)	0.02 (0.01, 0.03) *	0.051 (0.009, 0.093)	0.00 (0.00, 0.01) ^+^

^+^ 95% CI proportion of variance did include zero. * 95% CI proportion of variance between lower 0.01 and upper 0.08. ** 95% CI proportion of variance between lower 0.08 and upper 0.21.
